# Genetic diversity of murine norovirus associated with ethanol sensitivity

**DOI:** 10.1007/s00253-025-13410-8

**Published:** 2025-01-28

**Authors:** Aken Puti Wanguyun, Wakana Oishi, Daisuke Sano

**Affiliations:** 1 Department of Frontier Science for Advanced Environment, Graduate School of Environmental Studies, Tohoku University, Sendai, Japan; 2 Department of Civil and Environmental Engineering, Graduate School of Engineering, Tohoku University, Sendai, Japan

**Keywords:** Murine norovirus, Ethanol, Disinfection, Sensitivity, Genetic diversity

## Abstract

**Abstract:**

RNA viruses have high genetic diversity, allowing rapid adaptation to environmental pressures, such as disinfection. This diversity increases the likelihood of mutations influencing the viral sensitivity to disinfectants. Ethanol is widely used to control viral transmission; however, insufficient disinfection facilitates the survival of less-sensitive viruses. Further, the underlying mechanisms of ethanol-induced changes in viral sensitivity remain unclear. Here, we assessed the genetic characteristics of ethanol-adapted murine norovirus (MNV) and associated changes in viral sensitivity. Experimental ethanol-facilitated MNV adaptation and subsequent genetic characteristic evaluation of the whole genome sequence was performed. MNV was exposed to 70% ethanol for 5 s to achieve ± 3-log_10_ inactivation. Twelve MNV populations were identified as “less sensitive,” consisting of nine treated and three control populations. Less-sensitive MNV populations exhibited significantly higher synonymous nucleotide diversity (πS) in ORF1 (*p* = 0.001), which encodes the non-structural protein, than sensitive populations. Ethanol sensitivity and πS were negatively correlated in ORF1 (*R* = − 0.49, *p* = 0.003), indicating that high genetic diversity in ORF1 could be linked to reduced ethanol sensitivity. This study demonstrates an association between nucleotide diversity in specific coding regions of the MNV genome and ethanol sensitivity. These findings are vital for improving disinfection methods and anticipating emerging viruses that are more resistant to disinfectants.

**Key points:**

• *Several MNV populations reduced sensitivity to ethanol*.

• *Higher synonymous diversity in ORF1 linked to reduced ethanol sensitivity*.

• *Synonymous mutations can influence viral adaptation to ethanol*.

**Supplementary Information:**

The online version contains supplementary material available at 10.1007/s00253-025-13410-8.

## Introduction

The World Health Organization (WHO) has identified enteric viruses as the leading cause of acute gastroenteritis, significantly impacting global mortality and morbidity, particularly among children (Troeger et al. 2017). Among these enteric viruses, some belong to the RNA virus group (Aggarwal et al. 2021). RNA viruses are characterized by rapid generation times, high mutation rates, and significant genetic diversity due to the absence of a proofreading mechanism during replication (Sanjuán and Domingo-Calap 2021). Human noroviruses are single-stranded, positive-sense, non-enveloped RNA enteric viruses that commonly cause acute gastroenteritis (Chhabra et al. 2019). They are classified into at least 10 genogroups (GI-GX) and 48 genotypes (Chhabra et al. 2019). Noroviruses are environmentally stable and can be transmitted through the fecal–oral route (Pogan et al. 2018). Noroviruses can proliferate in the salivary glands and are transmitted through saliva (Ghosh et al. 2022). Transmission can also occur via environmental surfaces, such as doorknobs and toilet seats, creating opportunities for infection in the absence of proper hygiene practices (Alidjinou et al. 2019).

Effective disinfection methods are necessary to limit viral spread and prevent infections. To enhance preventive measures, the WHO recommends improving hygiene through regular handwashing or the use of alcohol-based disinfectants, such as ethanol, for daily hand hygiene (Ghafoor et al. 2021). Ethanol disinfection is frequently applied to surfaces in public places and healthcare facilities. Ethanol inactivates enveloped viruses by effectively dissolving lipid membranes, leading to the disassembly of virus particle structures (Ali et al. 2021). In non-enveloped viruses, ethanol modifies the structure or intermolecular interactions within viral proteins, promoting protein coagulation within the viral capsid (Ali et al. 2021). While enveloped viruses are generally susceptible to ethanol, the susceptibility of non-enveloped viruses varies (Wanguyun et al. 2023).

The efficacy of viral inactivation depends on appropriate disinfection conditions, including concentration and contact time, which vary depending on the specific virus involved (Lin et al. 2020). Sensitivity to disinfectants varies among viruses and even among highly related strains (Wanguyun et al. 2023). For instance, Ijaz et al. (2021) demonstrated differences in the efficacy of various disinfectants against SARS-CoV-2 and other coronavirus strains (Ijaz et al. 2021). Another study on norovirus inactivation using various antiseptic agents demonstrated varying sensitivities among genogroups and genotypes (Imai et al. 2020). The mechanisms of adaptation to disinfectants vary depending on the virus type and the disinfectant used (Oishi et al. 2022; Kadoya et al. 2022; Wanguyun et al. 2024).

Analyzing the genetic traits of viral populations exposed to disinfectants can provide valuable insights into how viruses evolve and adapt to specific disinfectants. A previous study found that murine norovirus (MNV) exposed multiple times to an initial free chlorine concentration of 50 ppm showed reduced sensitivity and a significant decrease in synonymous nucleotide diversity within major capsid proteins (Wanguyun et al. 2024). In contrast, rotaviruses repeatedly exposed to chlorine disinfection exhibited stochastic changes in sensitivity, accompanied by an increase in nonsynonymous nucleotide diversity (Kadoya et al. 2022). Another study observed that a specific mutation in the major capsid protein of MNV populations adapted to calcium hydroxide was associated with reduced susceptibility (Oishi et al. 2022). Given the potential alteration in disinfectant sensitivity in viruses, clarifying the factors that lead to changes in disinfectant susceptibility by understanding the genetic characteristics of viral populations is essential.

In this study, we hypothesized that ethanol-adapted virus populations exhibit specific variations influencing their ethanol sensitivity, which can be identified using genome sequencing. Therefore, the objective of this study was to elucidate the effects of ethanol exposure on the genetic traits of virus populations associated with changes in virus sensitivity to ethanol based on whole genome sequencing. By understanding the genetic alterations between less-sensitive and sensitive virus populations, we can gain deeper insights into how more resistant viral populations can emerge in response to disinfectant exposure.

## Materials and methods

### Virus propagation

MNV strain S7 was cultured in mouse macrophage-like RAW 264.7 cells (ATCC TIB-71). The cells were maintained in Dulbecco’s Modified Eagle’s Medium (DMEM) supplemented with 10 mM nonessential amino acids, 0.1% (w/v) NaHCO_3_, HEPES, 10% fetal bovine serum (FBS, Gibco), 100 mg/mL penicillin, 100 U/mL streptomycin, and 2 mM L-glutamine. When cell monolayers reached approximately 80% confluence, they were inoculated with MNV and incubated at 37 °C with 5% CO_2_ for approximately 3 days until cytopathic effects were observed.

### Serial passage

**Fig. 1:**
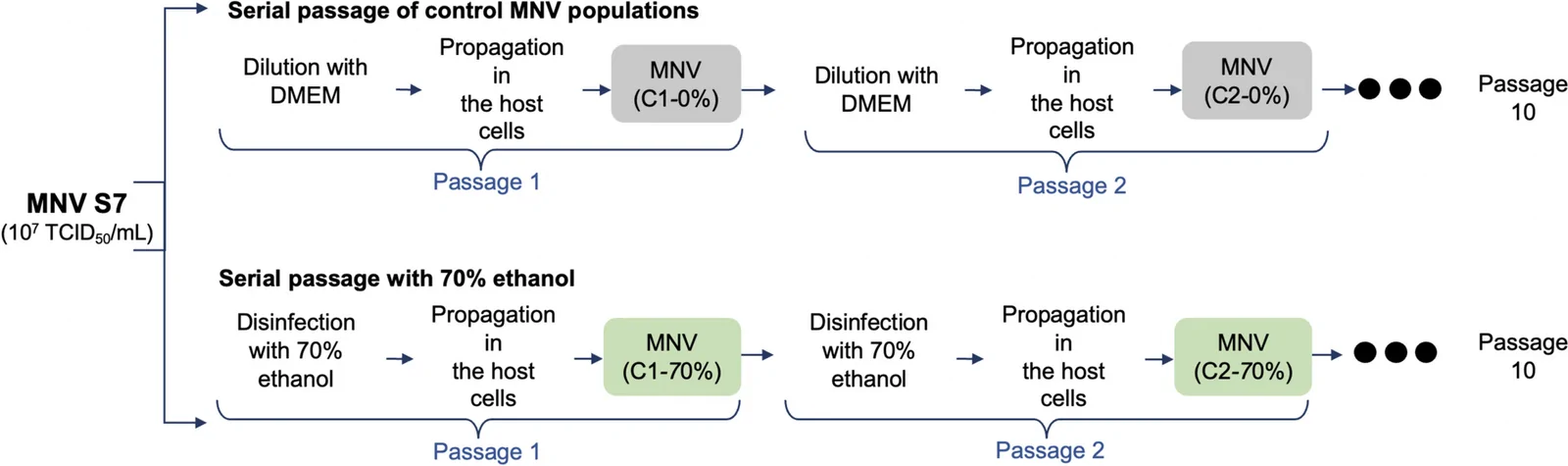
Diagrammatic illustration of the two conditions during serial passage experiments

A wild-type MNV S7 suspension (titer: 10^7^ TCID_50_/mL) was prepared, which was cultured in RAW 264.7 cell monolayers and used as host cells. MNV was used for serial passage experiments under several conditions to constitute the control and ethanol-treated populations. MNV populations obtained through dilution from the *x*th passage were treated with ethanol at concentrations of 0% and 70% and were designated as C*x*−0% and C*x*−70% virus, respectively (*x* = 1, 2, 3, 4, 5, 6, 7, 8, 9, 10) (Fig. 1). The serial MNV passage experiment was conducted in duplicate (rounds 1 and 2) for each population.

Ethanol (70%) was prepared by adding absolute ethanol to phosphate-buffered saline. C*x*−70% MNV suspensions (10^7^ TCID_50_/mL) were exposed to 70% ethanol for 5 s to inactivate approximately 3 orders of magnitude of the viral titer, as the minimum required level of inactivation after disinfection based on US Environmental Protection Agency guidelines (USEPA 2020). Cold DMEM containing 10% FBS was then used for neutralization (Escudero-Abarca et al. 2022). Simultaneously, C*x*−0% was obtained by diluting the MNV with DMEM to prepare a 10^3 ^TCID_50_/mL solution. RAW 264.7 cells with approximately 10^−5^ multiplicity of infection were cultured and incubated at 37 °C in a 5% CO_2_ atmosphere for approximately 72 h. Following regrowth, the virus–cell suspensions were subjected to three freeze–thaw cycles to release the viral particles from the cells. The supernatant was centrifuged at 10,000 × g for 30 min at 4 °C, followed by filtration through a 0.45-μm polyvinylidene difluoride membrane filter to eliminate cellular debris. The entire experiment was performed in duplicate to ensure reproducibility. The resulting viral populations were stored at − 80 °C for further analysis.

### Determination of virus sensitivity to ethanol

The ethanol sensitivity of the MNV populations, both treated and untreated with disinfection, was evaluated at each serial passage cycle by measuring the reduction of viral titers following exposure to 70% ethanol. The viral sensitivity test was performed by mixing 50 μL of MNV suspension with 450 μL of 70% ethanol and incubating for 5 s. This was followed by neutralization via a tenfold dilution with cold DMEM containing 10% FBS. Samples from each cycle were evaluated in triplicate. The TCID_50_ (50% tissue culture infectious dose) assay was used to measure viral concentration, as described in a previous study (Lei et al. 2021). TCID_50_ results were used to determine the log_10_ reduction value (LRV), indicating the reduction in viral concentration after disinfection and expressed in logarithmic units. LRV was calculated as the ratio of the initial virus concentration to the final concentration after treatment (Log_10_ (*Nt*/*N*_0_)).

### Library preparation and whole genome sequencing

Viral samples collected from the serial passage experiments were subjected to library preparation and whole-genome sequencing. RNA was extracted from the viral suspension using the QIAamp Viral RNA Mini kit (Qiagen, Hilden, Germany) according to the manufacturer’s protocol. Prior to library preparation, ribosomal RNA (rRNA) was depleted using the NEBNext rRNA Depletion Kit (human/mouse/rat; New England Biolabs, Ipswich, MA, USA). Libraries were then prepared using the NEBNext Ultra II RNA Library Prep kit (Illumina) and NEBNext Multiplex Oligos (Illumina), following the manufacturer’s instructions. The library was purified using AMPure XP magnetic beads (Beckman Coulter, Pasadena, CA, USA). Quality control included assessment of library size distribution and quantification using an Agilent Bioanalyzer, HS DNA Assay kits, and a Qubit 2.0 fluorometer (Invitrogen, Carlsbad, CA, USA). The final library concentration was measured using qPCR with the NEBNext Library Quant kit for Illumina before sequencing, which involved paired-end 150-bp reads on an Illumina NovaSeq 6000 (Illumina, San Diego, CA, USA).

### Bioinformatics analysis of the sequencing libraries

Bioinformatics analysis was performed using CLC Genomics Workbench 10.1.1 (CLC Bio, Aarhus, Denmark). Initially, quality trimming and barcode sequence removal were performed. The *Trim Sequences* option was used to remove adaptors. The resulting sub-reads were aligned against the reference genome of MNV S7 (GenBank: AB435515.1) using the Map Reads to the Reference option in CLC Genomics Workbench. The *Reference Mapping* option was used to assemble the trimmed reads into reference sequences, which were subsequently realigned using the *Local Realignment* function.

The consensus sequence was derived from ancestral MNV populations in the serial passage experiments and used as a reference sequence for detecting variants using the *Extract Consensus Sequence* function. Single-nucleotide polymorphism (SNP) frequency was computed using the *Low Frequency Variant Detection* function with an error rate threshold set at 1%. SNPs with coverage depths less than 10 were filtered out using the default configuration of CLC Genomics Workbench. The potential impact of SNPs on amino acid sequences was assessed using the *Amino Acid Changes* feature for each open reading frame (ORF) in the MNV genome, including ORF1, which encodes a non-structural protein, ORF2, which encodes the major capsid protein (VP1), and ORF3, which encodes the minor capsid protein (VP2) (Barron et al. 2011). The organization of the MNV genome is illustrated in Supplementary Fig. S1.

### Nucleotide diversity analysis

Genetic diversity was analyzed within the virus populations by calculating the mean pairwise nucleotide diversity (π), representing the average number of pairwise differences per site in a sequence population (Nelson and Hughes 2015). The π value was determined using the following equation:

1 $$\pi = \sum_{i=1}^{p}Di/L$$

2 $$Di= \frac{AiCi+AiGi+AiTi+CiGi+CiTi+GiTi}{({c}_{i}^{2}-ci)/2}$$

where *p* is the number of polymorphic sites, *Di* is the nucleotide difference proportion at the *i*th site, and *L* is the sequence length; *ci* is the coverage at the *i*th site; and *Ai*, *Ci*, *Gi*, and *Ti* are the counts of the four bases at the *i*th site (Nelson et al. 2015; Kadoya et al. 2022). Nucleotide diversity in nonsynonymous and synonymous coding sites (πN and πS) was determined using SNPGenie, reflecting the average number of differences between pairs of nucleotide positions in a sequence population (Nelson et al. 2015).

### Statistical analysis

Statistical analysis was conducted using the GraphPad Prism software (Version 10.1.1, 2023). The statistical significance of differences between two groups was assessed using the Mann–Whitney *U* test. All statistical tests were two-tailed, with a *p*-value of less than 0.05 considered significant. Results were visualized using Python 3.11 and RStudio (Version 2023.06.0).

## Results

### Sensitivity of MNV populations to ethanol

**Fig. 2:**
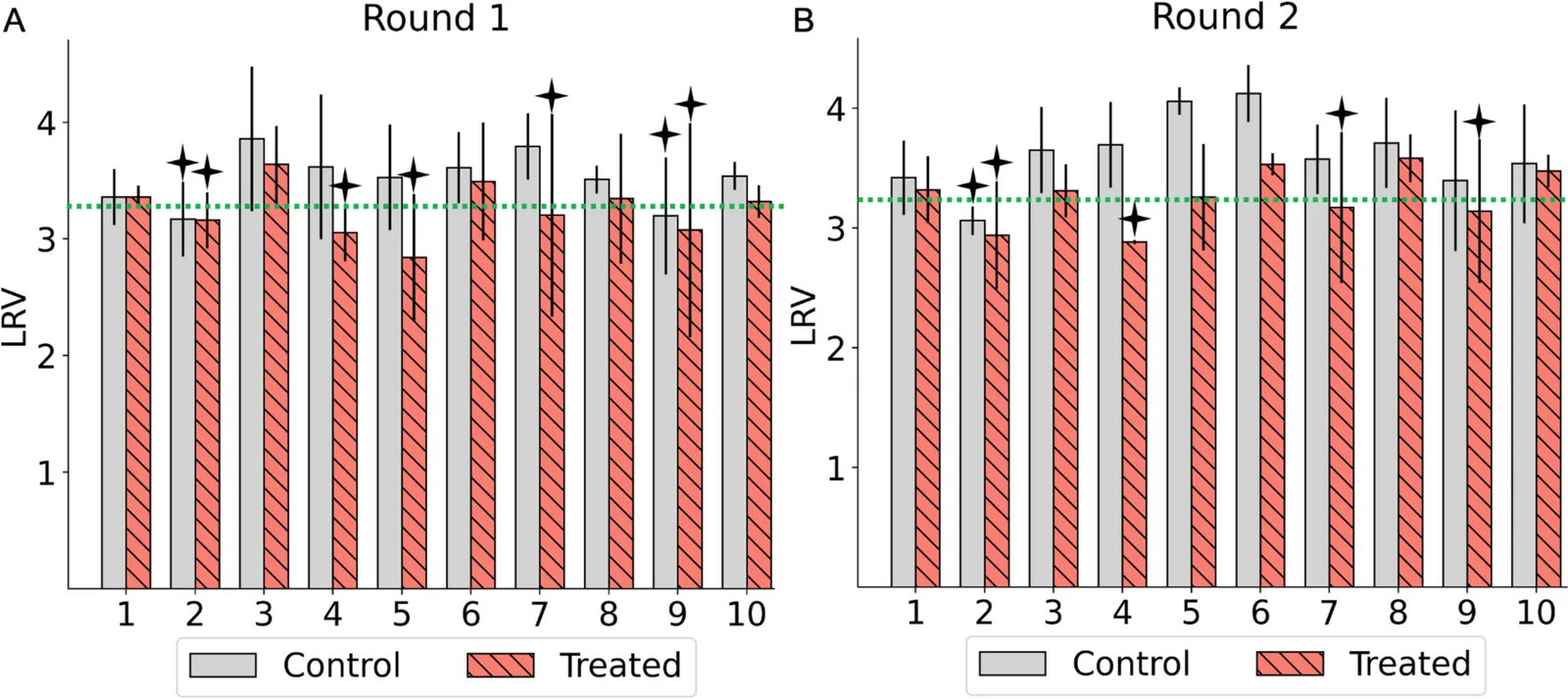
Log_10_ reduction value (LRV) of control and treated MNV populations from the first to the tenth passages in round 1 (**A**) and round 2 (**B**). The green dashed line indicates the median LRV of the control population (0 ppm) in the first passage of each round. Four-point stars above the bars highlight the less-sensitive populations. Each bar represents the average LRV from three replicates, with standard deviation

Figure 2 illustrates the sensitivity of MNV populations to ethanol from the first to the tenth passage in both control and treated groups. Ethanol (70%) application with 5 s contact time resulted in an average reduction of 3.23-log_10_ ± 0.21. Overall, the treated MNV populations exhibited a lower average log reduction value over the course of 10 passages compared to the control populations. Throughout all passages, there was a change in sensitivity, with treated MNV populations showing a tendency towards decreased sensitivity, although this change was not statistically significant (*p* > 0.05). A 3.34-log_10_ inactivation threshold was considered the cutoff value of LRV to determine MNV populations that were less sensitive to ethanol exposure. This threshold was based on the average LRV of the control population in the first passage of both rounds. We also used the Wilcoxon signed-rank test to identify virus populations that were less sensitive to ethanol, with the null hypothesis that the LRV of the MNV populations was equivalent to the cutoff value of the control populations in each round. Twelve MNV populations with LRV values below the cutoff value were considered less sensitive to ethanol. This group comprised seven populations from round 1 and five populations from round 2. In round 1, the less-sensitive populations included two control populations and five treated populations, whereas in round 2, there was one control population and four treated populations. The remaining viral populations were categorized as sensitive.

### Genetic diversity analysis among MNV populations

**Fig. 3:**
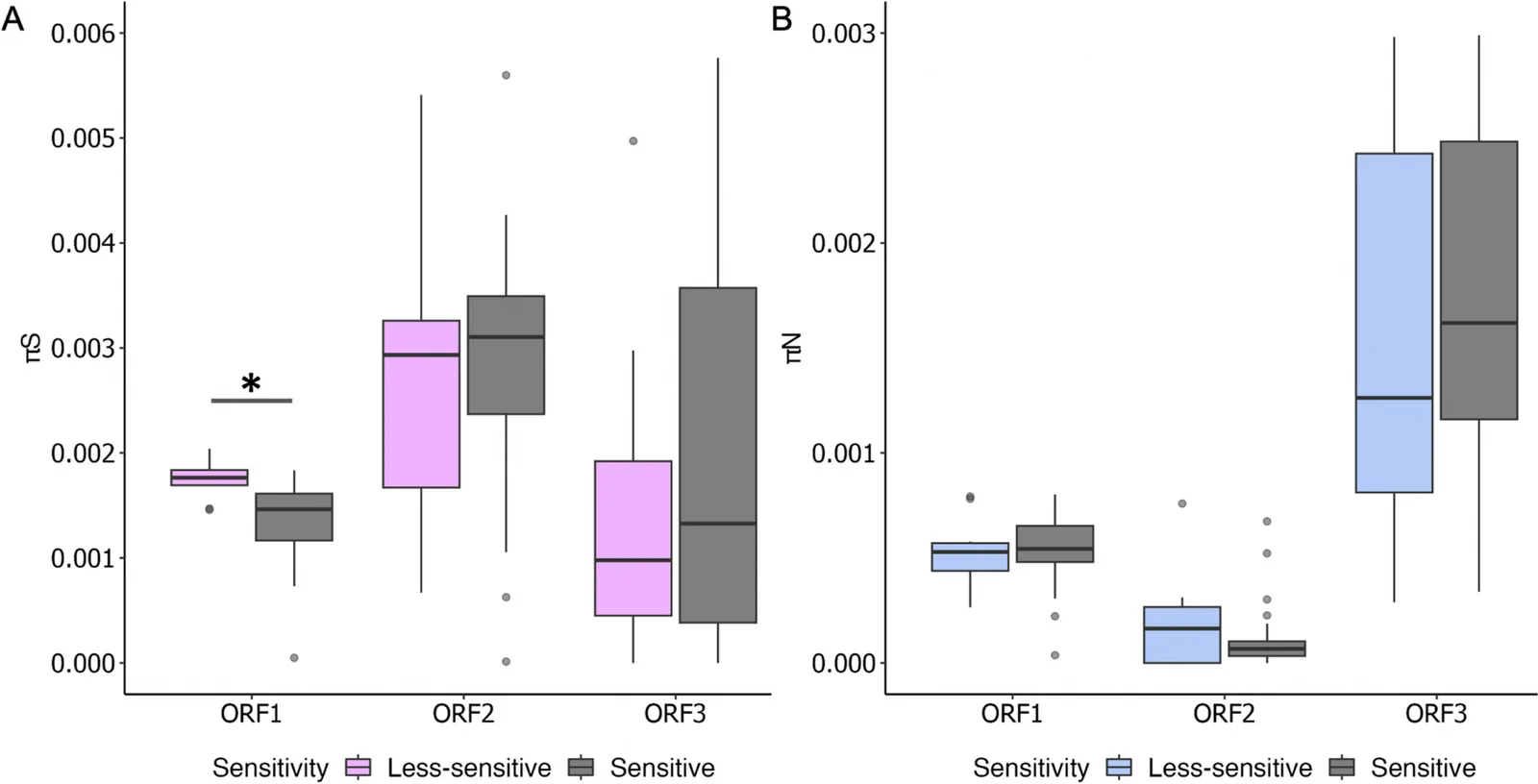
Average synonymous nucleotide diversity (πS) (**A**) and nonsynonymous nucleotide diversity (πN) (**B**) of MNV in each coding region. An asterisk indicates *p* < 0.05

**Fig. 4:**
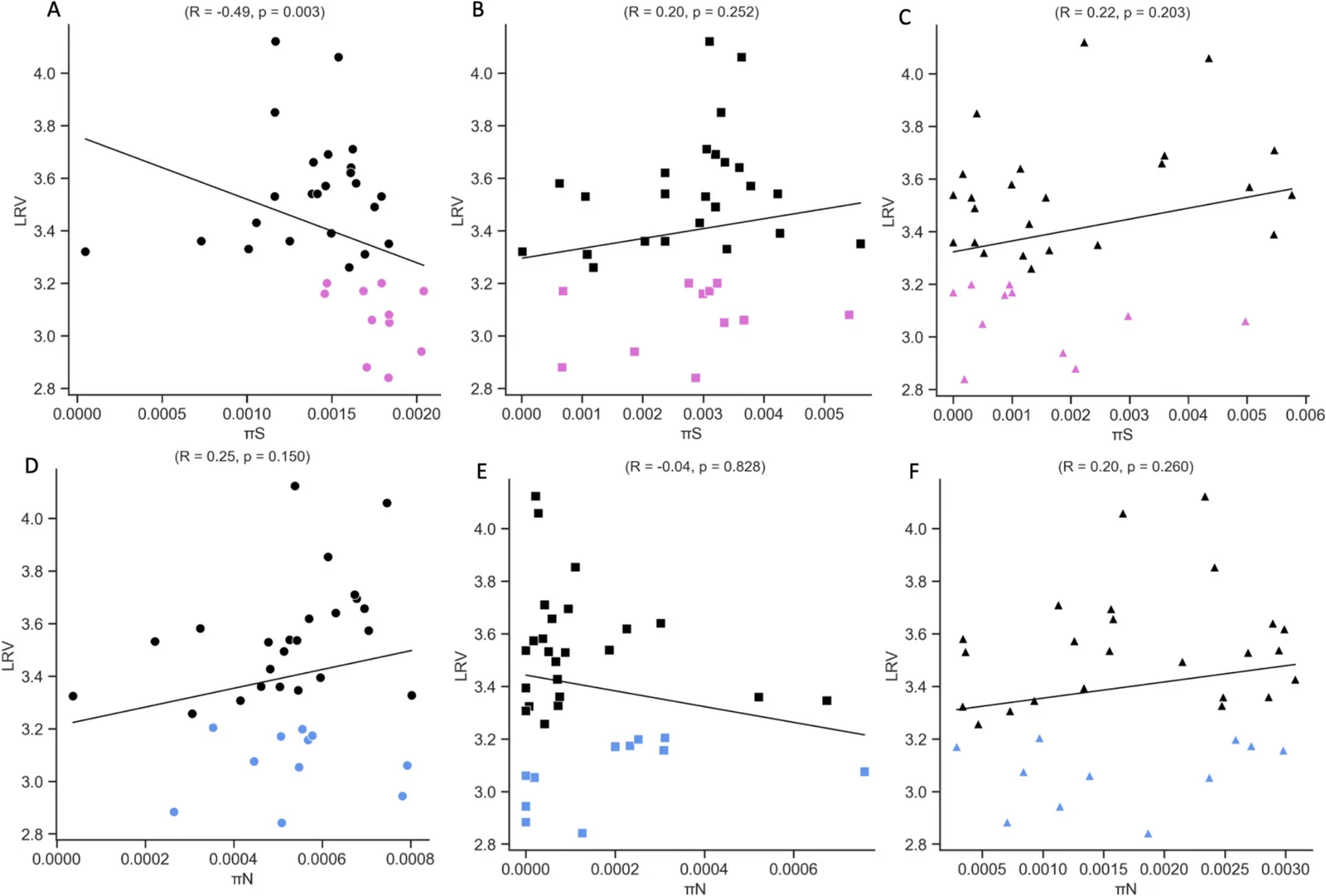
Correlation between log_10_ reduction value (LRV) and synonymous nucleotide diversity (πS) (**A**, **B**, **C**) and nonsynonymous nucleotide diversity (πN) (**D**, **E**, **F**) based on Spearman’s correlation coefficient (R) and *p*-value in ORF1, ORF2, and ORF3. Less-sensitive populations are colored magenta in πS and blue in πN, while sensitive populations are colored black

The evolution and adaptation of viruses are driven by the genetic diversity within viral populations. We identified synonymous nucleotide diversity (πS) and nonsynonymous nucleotide diversity (πN) in each coding region of the MNV genome for both less-sensitive and sensitive populations (Figs. 3 A and B). The average πS of the less-sensitive MNV populations was significantly higher in ORF1 than that in the sensitive populations (Mann–Whitney *U* test, *p* = 0.001). The mean πN value for VP1 in less-sensitive populations was higher than that in the sensitive populations, although this difference was not statistically significant (Mann–Whitney *U* test, *p* > 0.05). To further analyze the evolutionary forces shaping viral populations, the ratio of nonsynonymous to synonymous nucleotide diversity (πN/πS) was evaluated in each coding region (Zhao and Illingworth 2019). A πN/πS ratio greater than one indicated positive selection, suggesting an excess of nonsynonymous polymorphisms. Conversely, a ratio less than one indicated purifying selection, signifying an excess of synonymous polymorphisms (Zhao and Illingworth 2019). When the πN/πS ratio is approximately equal to one, neutral evolution is indicated (Zhao and Illingworth 2019). The average πN/πS ratios in ORF1 and ORF2 were less than one, indicating purifying selection, while the ratio in ORF3 exceeded one, indicating positive selection (Supplementary Fig. S2). The average πN/πS ratio in ORF3 of the less-sensitive populations was higher than that of the sensitive populations, although this difference was not statistically significant (Mann–Whitney *U* test, *p* > 0.05). Additionally, the average πS exceeded πN in all coding regions. Spearman’s correlation coefficient (*R*) was also calculated to examine the statistical association between ethanol sensitivity (LRV) and both synonymous and nonsynonymous nucleotide diversity in each coding region (Fig. 4). The correlation between πS and LRV was significantly negative only in ORF1 (*R* = − 0.49,* p* = 0.003).

### Identification of SNPs

**Fig. 5:**
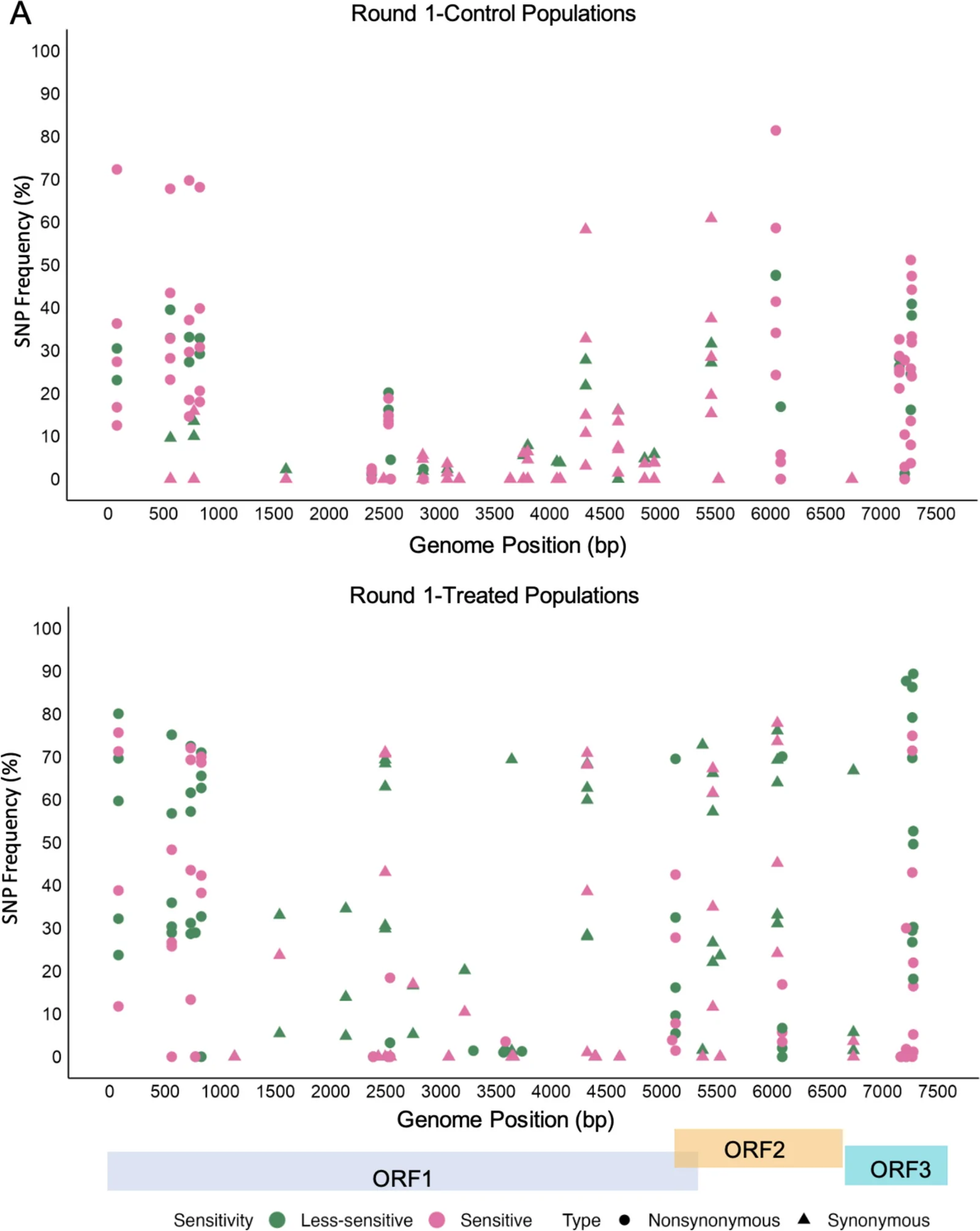

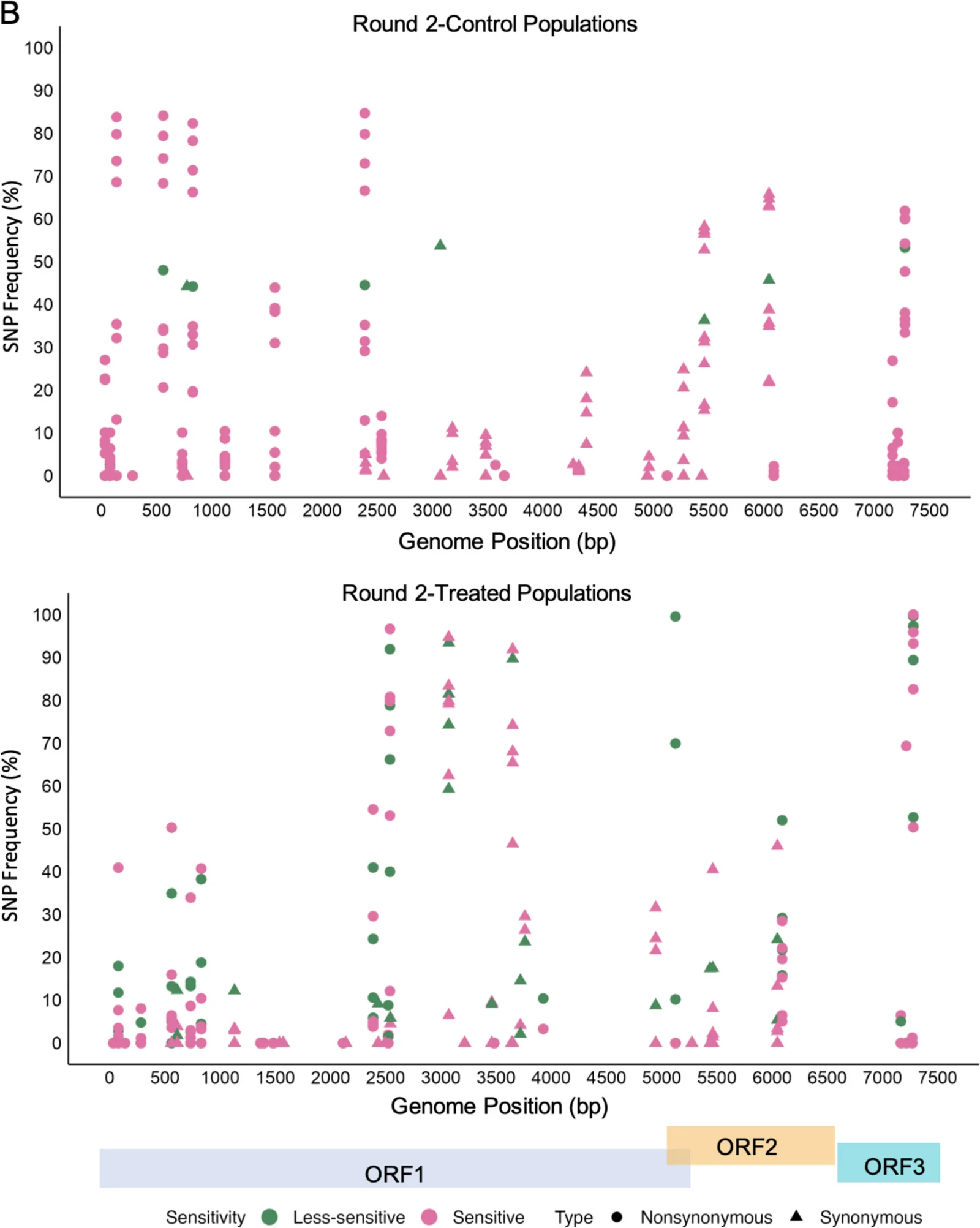
Distribution of SNP frequency (%) in control and treated populations in round 1 (**A**) and round 2 (**B**), colored by sensitivity and shaped by mutation types. Coding regions of the MNV genome are shown at the bottom of the figure

**Fig. 6:**
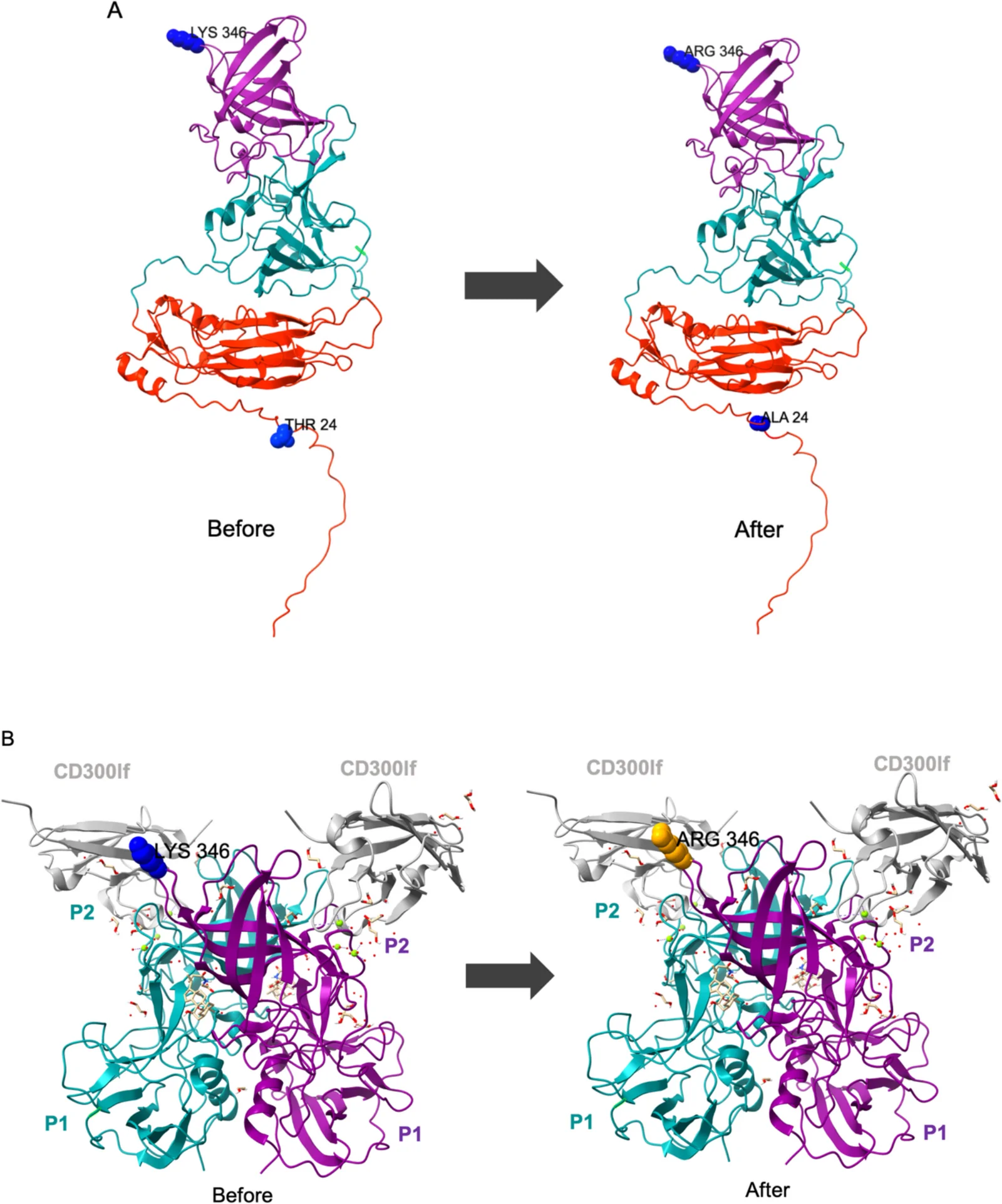
Conformational mapping of nonsynonymous mutations in the major capsid protein (VP1) of MNV before and after exposure to ethanol. S is shaded in red, P1 in teal, and P2 in purple (A). Amino acid substitutions in VP1 are highlighted with blue spheres, indicating the alteration of threonine (THR) to alanine (ALA) and lysine (LYS) to arginine (ARG), denoted as T24A and K346R, respectively. Amino acid alterations within the P domain, previously denoted as K346R, are shown as blue spheres (left) and after substitutions, illustrated as orange spheres (right) on the ribbon model of the CD300lf-P domain complex structure (B) (Nelson et al. 2018). The two chains constituting the P domain dimer are shaded teal and purple, with the CD300lf receptor protein displayed in silver

To identify specific mutations contributing to changes in ethanol sensitivity, the frequency of SNPs was analyzed in both control and treated MNV populations across all rounds (Fig. 5). No specific mutations were linked to reduced ethanol sensitivity. However, there was an increase in synonymous mutations starting at the approximate 500-bp position and extending through the rest of the genome, particularly in ORF1, in both control and treated populations across all rounds. The most notable distinction was that synonymous mutations were frequent in treated MNV populations. Only ORF3 exhibited a greater number of nonsynonymous mutations than synonymous mutations, with the frequency of some mutations exceeding 90% in both control and treated populations. A detailed summary of nucleotide and amino acid alterations is provided in Supplementary Table S1. Some nonsynonymous mutations in the VP1 of MNV populations exposed to ethanol were identified at positions 5125 and 6092, with frequencies ranging from 5 to 90% in each viral population. These mutations resulted in threonine-to-alanine and lysine-to-arginine substitutions (T24A and K346R, respectively) in the S- and P-domains (Fig. 6A). Further mapping of nucleotide changes in the P domain of MNV VP1 complexed with its cellular receptor (CD300lf) revealed the presence of K346R in the P2 subdomain (Fig. 6B) (Goddard et al. 2018; Nelson et al. 2018; Meng et al. 2023). Moreover, a nonsynonymous mutation at position 7279 in all control and ethanol-treated populations resulted in the substitution of phenylalanine with serine in VP2 (F200S).

## Discussion

To the best of our knowledge, this is the first study to investigate how MNV populations adapt to ethanol exposure by assessing their genetic characteristics using whole-genome sequencing. Our aim was to elucidate the mechanisms underlying the formation of viral populations that exhibit reduced sensitivity to disinfectants through experimental adaptation. The results showed changes in sensitivity across all passages, with treated MNV populations exhibiting a low average LRV, indicating a reduction in sensitivity. Twelve MNV populations demonstrated LRV values below 3.34-log_10_, derived from seven populations in the first round and five in the second round (Fig. 2). The mean synonymous nucleotide diversity (πS) of less-sensitive populations was significantly higher than that of sensitive populations in ORF1 (Mann–Whitney *U* test, *p* = 0.001) (Fig. 3). Moreover, the correlation between πS and LRV was significantly negative only in ORF1 (*R* = − 0.49, *p* = 0.003) (Fig. 4). No significant differences were observed in πN across all coding regions. Synonymous mutations were prevalent throughout the genome, particularly in ORF1, in both control and treated populations across both rounds. However, the frequency of synonymous mutations was higher in the treated MNV populations than that in the control populations (Fig. 5). These results suggest that alterations in ethanol sensitivity in MNV populations can be attributed to variations in genetic traits within specific coding regions.

The sensitivity of viral populations to disinfectants depends on various factors, including the type of disinfectant, its concentration, contact time, and application conditions. One study highlighted that the key variables influencing the effectiveness of viral inactivation, particularly with ethanol, can differ depending on the type of virus (Wanguyun et al. 2023). Alterations in viral sensitivity to disinfectants are associated with mechanisms, such as specific mutations or changes in genetic diversity (Carratalà et al. 2017, 2020; Oishi et al. 2022; Kadoya et al. 2022; Wanguyun et al. 2024). Our findings indicated that the less-sensitive MNV populations had a significantly higher πS in ORF1 than the sensitive populations, with a significant negative correlation between πS and LRV. These results align with a previous study that found a significant negative correlation between πS and LRV in ORF1 of MNV populations adapted to calcium hydroxide (Oishi et al. 2022). ORF1 encodes a single polyprotein that is subsequently cleaved into six non-structural proteins (NSPs) (NS1/2, NS3, NS4, NS5, NS6, and NS7). These NSPs are crucial for membrane rearrangements and essential for norovirus replication within host cellular membranes (Doerflinger et al. 2017). Although synonymous mutations do not alter amino acids, they can affect RNA structural characteristics (Tubiana et al. 2015). Synonymous mutations can alter the mRNA structure, create novel promoters, and enhance gene functionality under selection (Kershner et al. 2016; Lebeuf-Taylor et al. 2019). Furthermore, synonymous mutations can increase the availability of aminoacyl-tRNA molecules, improving protein translation efficiency and potentially leading to efficient viral particle translation (Alonso and Diambra 2020; Ramazzotti et al. 2022). While synonymous mutations are generally considered neutral regarding fitness, the variability in their effects remains unclear (de Jong et al. 2024). Further analysis is necessary to understand the mechanisms by which synonymous mutations affect viral fitness and potentially enhance viral adaptation.

Some non-synonymous mutations appeared in all MNV populations exposed to ethanol in ORF2 at positions 5125 and 6092. However, the presence of these mutations, even in the treated MNV populations that did not show reduced sensitivity to ethanol, indicates that this mutation is not the sole factor responsible for the change in sensitivity. Although these mutations are not the primary cause of reduced viral sensitivity, the amino acid changes in VP1 have the potential to affect the essential functions of viral capsids (Campillay-Véliz et al. 2020). ORF2, which encodes VP1, consists of a shell (S) domain and a protruding (P) domain, further subdivided into proximal (P1) and distal (P2) subdomains connected by a flexible hinge region (Nelson et al. 2018). The S domain forms the interior surface that protects the viral capsid within the icosahedral shell, while the P domain protrudes upward from the S domain and mediates interactions with receptor molecules (Snowden et al. 2020). Furthermore, the P1 subdomain forms a stem connecting the S domain to the globular head region, and the P2 subdomain is the outermost part of the viral capsid, functioning as a viral attachment site to cells (Katpally et al. 2008). Within the S domain, an amino acid alteration was identified, resulting in a change from threonine to alanine. This shift from a polar to a nonpolar amino acid could potentially affect the protein structure. One study suggested that mutations in the shell domain may not cause significant structural shifts but can induce small changes that influence antibody binding in norovirus (Zhu et al. 2016). An amino acid substitution, K346R, was observed in the P2 subdomain (Fig. 6). The P2 region of the P domain contains receptor binding site, serving as the site for viral attachment to host cells (Nelson et al. 2018). Disruption of the site-specificity of the viral capsid protein can also enhance capsid disassembly, leading to increased genome release (Ghaemi et al. 2021). A previous study found that MNV populations with significantly reduced sensitivity to chlorine exhibited a higher number of non-synonymous mutations in both the P1 and P2 subdomains compared to the chlorine-sensitive MNV populations (Wanguyun et al. 2024). However, further investigations, such as enzyme-linked immunosorbent assay or binding polymerase chain reaction, are required to confirm the specific mechanism by which these mutations affect the virus–receptor interaction. Moreover, the observed non-synonymous mutation at position 7279 of VP2 in both control and treated MNV populations is consistent with the findings of previous studies (Oishi et al. 2022; Wanguyun et al. 2024). VP2 plays several important roles in the replicative cycle and modulates immune responses (Campillay-Véliz et al. 2020).

The findings of this study provide valuable insights into the mechanisms by which viral populations adapt to rapid disinfection, expanding our understanding beyond the scope of previous studies that have primarily focused on disinfectants used for water treatment (Carratalà et al. 2017, 2020; Kadoya et al. 2022; Wanguyun et al. 2024). Although the use of MNV as a surrogate for human norovirus may present some limitations, this study offers important perspectives on viral sensitivity to disinfection. MNV is a valuable surrogate due to its close genetic relationship with human noroviruses, similar genome structure, and common transmission mode, despite differences in host specificity and some molecular interactions (Belliot et al. 2008; Tan and Jiang 2010). Therefore, the insights gained from this study can be extended to human noroviruses (Le Pendu et al. 2016; Graziano et al. 2020).

Analyzing the genetic composition of viral populations provides valuable information on the mechanisms through which these populations adapt to disinfection. In the less-sensitive MNV populations, genetic alterations primarily occurred within ORF1 and were associated with reduced viral sensitivity to ethanol. Our findings highlight that variations in specific coding regions of the viral genome affect viral sensitivity. Additionally, the presence of synonymous mutations in ORF1 in the less-sensitive populations may contribute to viral disinfectant adaptation. Therefore, further investigating the specific mechanisms by which ORF1 influences viral adaptation is crucial. The findings not only deepen our understanding of viral adaptation to disinfection but also suggest potential applications of this understanding in enhancing the control of viral infections in healthcare settings.

## Supplementary Information

Below is the link to the electronic supplementary material.

Supplementary file1 (PDF 347 KB)

## Data Availability

The sequences described in this study are available in GenBank under accession numbers PP550055–PP550090.
