# Supplementary material for: Genetic diversity of murine norovirus associated with ethanol sensitivity
Source: Appl Microbiol Biotechnol. 2025 Jan 28;109(1):28. doi: 10.1007/s00253-025-13410-8 (PMC11775025; doi:10.1007/s00253-025-13410-8)
Supplement: Supplementary file 1 — Supplementary file1 (PDF 347 KB) [file 253_2025_13410_MOESM1_ESM.pdf]

Supplementary Material

Genetic Diversity of Murine Norovirus Associated with Ethanol Sensitivity

Aken Puti Wanguyun[1], Wakana Oishi[2], Daisuke Sano[1,2,*]

[1]Department of Frontier Science for Advanced Environment, Graduate School of Environmental Studies, Tohoku University, Sendai, Japan

[2]Department of Civil and Environmental Engineering, Graduate School of Engineering, Tohoku University, Sendai, Japan

*Corresponding author: Email: daisuke.sano.e1@tohoku.ac.jp

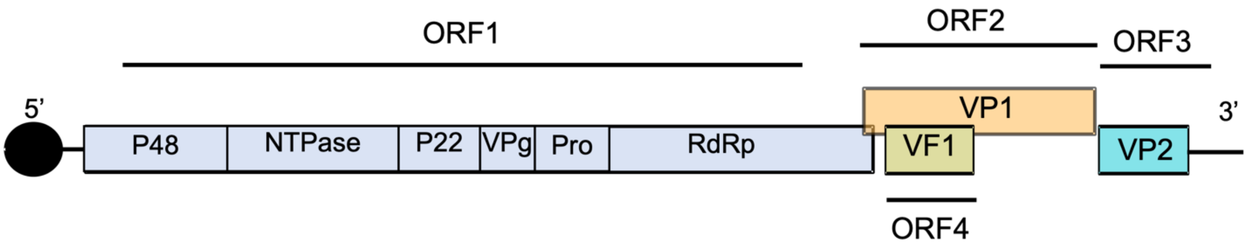

Figure S1. Schematic diagram illustrating the organization of the MNV genome. There were three main open reading frames (ORFs), encompassing ORF1 (nucleotides 6 to 5069), encoding a non-structural polyprotein, ORF2 (nucleotides 5056 to 6681), encoding a major capsid protein (VP1), and ORF3 (nucleotides 6681 to 7307), encoding a minor capsid protein (VP2) (Sosnovtsev et al. 2006; Barron et al. 2011; Zhang et al. 2023)

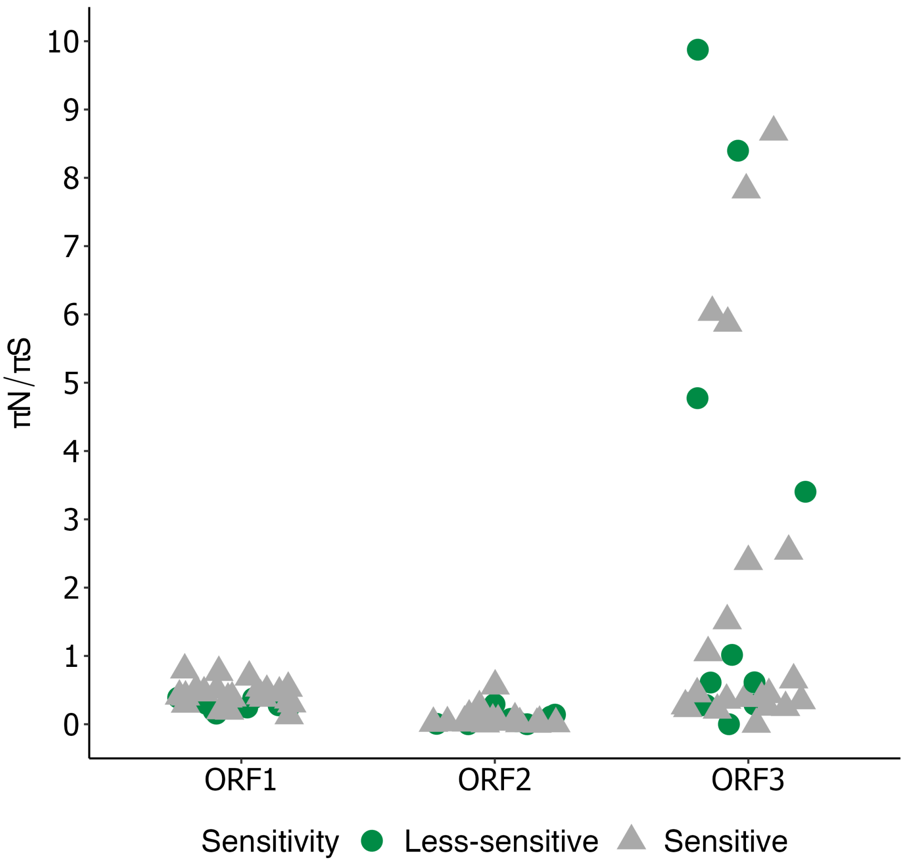

Figure S2. Values of πN/πS for ORF1, ORF2, and ORF3 across all MNV populations

Table S1. Changes in nucleotides and amino acids in the genomes of the MNV in the first round (A) and in the second round (B)

A

Coding Region	Round 1 Position	Amino Acid Change	Amino Acid Position
ORF1	T78C	S → P	25
	T561C	P → S	186
	A733G	Y → C	243
	C776T	No change	
	C829T	T → M	275
	T2386C	V → A	794
	T2495C	No change	
	T2523C	S → P	840
	A2539G	H → R	845
	C3641T	No change	
	C4325T	No change	
	C4619T	No change	
ORF2	T5095C	S → P	14
	A5125G	T → A	24
	C5370T	No change	
	C5463T	No change	
	C6048T	No change	
	A6092G	K → R	346
ORF3	T6737C	No change	
	T7167C	S → P	163
	C7215T	W → R	179
	T7270C	F → S	197
	T7279C	F → S	200

Note: A, alanine; R, arginine; C, cysteine; H, histidine; I, isoleucine; K, lysine; M, methionine; F, phenylalanine; P, proline; S, serine; T, threonine; W, tryptophan; Y, tyrosine; V, valine.

B

Coding Region	Round 2 Position	Amino Acid Change	Amino Acid Position
ORF1	T33C	S → P	10
	T78C	S → P	25
	G138A	A → T	45
	C283T	S → F	93

	T561C	P → S	186
	A733G	Y → C	243
	C829T	T → M	275
	A1120G	K → R	372
	G1572T	A → S	523
	T2386C	V → A	794
	A2539G	H → R	845
	T2558	No change	
	C2849T	No change	
	C3071T	No change	
	C3482T	No change	
	G3650A	No change	
	C4946T	No change	
ORF2	A5125G	T → A	24
	C5274T	No change	
	A5529G	No change	
	A5439G	No change	
	C5463T	No change	
	C6048T	No change	
	A6092G	K → R	346
	T6737C	No change	
ORF3	T7167C	S → P	163
	T7215C	W → R	179
	T7270C	F → S	197
	T7279C	F → S	200

Note: A, alanine; R, arginine; C, cysteine; H, histidine; I, isoleucine; L, leucine; K, lysine; M, methionine; F, phenylalanine; P, proline; S, serine; T, threonine; W, tryptophan; Y, tyrosine; V, valine.

References

Barron EL, Sosnovtsev S V., Bok K, Prikhodko V, Sandoval-Jaime C, Rhodes CR, Hasenkrug K, Carmody AB, Ward JM, Perdue K, Green KY (2011) Diversity of Murine Norovirus Strains Isolated from Asymptomatic Mice of Different Genetic Backgrounds within A Single U.S. Research Institute. PLoS One 6. https://doi.org/10.1371/journal.pone.0021435

Sosnovtsev S V., Belliot G, Chang K-O, Prikhodko VG, Thackray LB, Wobus CE, Karst SM, Virgin HW, Green KY (2006) Cleavage Map and Proteolytic Processing of the Murine Norovirus Nonstructural Polyprotein in Infected Cells. J Virol 80:7816–7831. https://doi.org/10.1128/jvi.00532-06

Zhang Q, Zhu S, Zhang X, Su L, Ni J, Zhang Y, Fang L (2023) Recent Insights into Reverse Genetics of Norovirus. Virus Res 325. https://doi.org/10.1016/j.virusres.2023.199046
